# Congenital Abnormalities and Multiple Sclerosis

**DOI:** 10.1186/1471-2377-10-115

**Published:** 2010-11-16

**Authors:** Sreeram V Ramagopalan, Colleen Guimond, Maria Criscuoli, David A Dyment, Sarah-Michelle Orton, Irene M Yee, George C Ebers, Dessa Sadovnick

**Affiliations:** 1 Wellcome Trust Centre for Human Genetics, University of Oxford, Oxford, OX3 7BN, UK; 2 Department of Clinical Neurology, University of Oxford, The West Wing, The John Radcliffe Hospital, Oxford, OX3 9DU, UK; 3 Department of Medical Genetics, University of British Columbia, G920, Detwiller Pavilion, VCHA - UBC Hospital, 2211 Wesbrook Mall, Vancouver, British Columbia, V6T 2B5, Canada; 4 Faculty of Medicine, Division of Neurology, University of British Columbia, G920, Detwiller Pavilion, VCHA - UBC Hospital, 2211 Wesbrook Mall, Vancouver, British Columbia, V6T 2B5, Canada

## Abstract

**Background:**

There is a strong maternal parent-of-origin effect in determining susceptibility to multiple sclerosis (MS). One hypothesis is that an abnormal intrauterine milieu leading to impaired fetal development could plausibly also result in increased susceptibility to MS. A possible marker for this intrauterine insult is the presence of a non-fatal congenital anomaly.

**Methods:**

We investigated whether or not congenital anomalies are associated with MS in a population-based cohort. We identified 7063 MS index cases and 2655 spousal controls with congenital anomaly information from the Canadian Collaborative Project on Genetic Susceptibility to MS (CCPGSMS).

**Results:**

The frequency of congential anomalies were compared between index cases and controls. No significant differences were found.

**Conclusions:**

Congenital anomalies thus do not appear to be associated with MS. However, we did not have complete data on types and severity of congenital anomalies or on maternal birth history and thus this study should be regarded as preliminary.

## Background

Multiple sclerosis (MS) is an inflammatory disease of the central nervous system (CNS) characterized by myelin loss, varying degrees of axonal pathology and progressive neurological dysfunction [1]. Autoimmune mechanisms are thought to have major roles in the pathogenesis of MS [2] but the cause of the disease is not yet conclusively understood [1]. A maternal parent-of-origin effect in MS [3,4] has now been well demonstrated but the biological basis underlying this remains unknown [5].

Approximately 3% of babies born in North America have a congenital anomaly [6]. A congenital anomaly is an abnormality of structure, function or body metabolism that is present at birth and results in physical or mental disability, with varying degrees of severity [7]. Purely genetic factors (chromosomes, single gene mutations) are believed to account for 15-25% of all congenital anomalies leaving up to 80% due to multifactorial inheritance or environmental exposures [6]. These latter two categories may be affected by factors such as fetal exposures to infection (e.g. cytomegalovirus, varicella, rubella), toxins (e.g. alcohol, cocaine, accutane) or maternal medical conditions (diabetes, epilepsy, phenylketonuria) [6]. It is possible that a compromised intrauterine environment underlies the maternal effect seen in MS. One hypothesis is that an abnormal intrauterine milieu leading to impaired fetal development could plausibly also result in increased susceptibility to MS. A possible marker for this intrauterine insult is the presence of a "non-fatal" congenital anomaly (hereafter referred to simply as a "congenital anomaly"). If this were true, one would expect an excess of congenital anomalies in MS patients compared to gender and ethnicity-matched controls.

## Methods

The population-based Canadian Collaborative Project on Genetic Susceptibility to Multiple Sclerosis (CCPGSMS) has been collecting perinatal data on MS index cases and spousal controls using structured telephone interviews with appropriate consents [7]. Data specific for the question of congenital anomalies were collected from mothers of index cases and mothers of controls. Each participating CCPGSMS site has obtained ethical approval from the relevant institutional review board. The entire project was reviewed and approved by the University of British Columbia in compliance with the Helsinki Declaration.

### Statistical analyses

The Chi squared test was used to assess significance when comparing the frequency of congenital anomalies in index cases and controls. The effect of congenital anomalies and sex on the risk of MS was assessed by logistic regression using the R statistical package.

## Results

Maternal information on congenital anomalies could be collected for 7063 MS index cases and 2655 spousal controls. The clinical and demographic details of the index cases and controls are shown in Table 1.

**Table 1:** Clinical and demographic details of MS index cases and controls

	MS Index Cases	Spousal Controls
**n**	7063	2655

**mean age in years (SD)**	50.2 (10.1)	52.3 (9.6)

**n (females)**	5319	807

**n (males)**	1744	1848

**sex ratio (f:m)**	3:1	0.4:1

**% relapsing remitting MS**	82.3	/

SD = standard deviation, (f:m) = female to male sex ratio

Two hundred and seventy (3.8%) MS index cases reported a congenital anomaly, not significantly different (*p *= 0.97) from controls (n = 101, 3.8%). As there is a preponderance of females with MS [8], there is by definition a corresponding over-representation of male spousal controls. If the trait of interest is sex-limited, then analysis by sex stratified cases and controls is important [9]. When we compared male index cases and female controls separately from male index case and male controls, there were again no significant differences. Logistic regression confirmed this showing an effect of female sex on the risk of MS (*p *< 1 × 10^-16^) but no effect of having a congenital abnormality (*p *= 0.92).

## Discussion

Environmental risk factors are known to be responsible for 8-12% of congenital anomalies and are a likely contributing factor in an additional 20-25% of anomalies as part of multifactorial inheritance [10,11] If the same risk factors acting *in utero *contribute to both congenital anomalies *and *future MS risk, then congenital anomalies could be used as biomarkers for MS. The large MS index case and control sample size used in this study did not support this hypothesis. It may well be possible that congenital anomalies are not good markers for intrauterine risk factors specific to MS or that the effect is too small to observe, given that only a small proportion of anomalies are attributed solely to environmental factors.

Our study does have limitations. Maternal recall of congenital anomalies is likely influenced by the time elapsed from the child's birth and by the severity of the condition and may not be as reliable as clinical records. For example, a mother would more likely recall a congenital anomaly needing medical intervention (e.g. cleft lip or palate, talipes) than one that may not (e.g. minor cardiac septal defect, spina bifida occulta). Conversely, a mother may over-report minor afflictions as congenital anomalies or they may report anomalies not detected at birth. The latter would help to explain the relatively high rate of anomalies observed compared to expected rates. As part of the CCPGSMS to date, we did not ask specifically for information on the type and severity of the congenital abnormality, which maybe important for MS risk. We also did not ask about the maternal history of fatal congenital abnormalities in previous pregnancies; this information would be missing from the family history especially if an affected pregnancy resulted in a termination, miscarriage, stillbirth, or perinatal death. We anticipate conducting a more focused questionnaire interview in the future as part of the ongoing CCPGSMS.

## Conclusions

The maternal effect in MS is substantial. Risk for MS in maternal half-siblings compared with the risk for full siblings does not differ significantly indicating that maternal effects might even be the major component of familial aggregation of the disease [4]. Our data suggests that congenital anomalies are not more prevalent among those who develop MS and seems to reduce the likelihood that there is a strong association between risk factors common to MS and congenital anomalies. However our study is a preliminary investigation as we did not have detailed data on congenital anomalies or maternal birth history. Further investigations are needed to explain the mechanisms underlying the increased MS risk conferred maternally.

## Compenting interests

The authors declare that they have no competing interests.

## Authors' contributions

GCE and ADS conceived and designed the experiments. SVR, CG, MC, DAD, SMO, IMY, GCE and ADS analyzed the data and wrote the paper. All authors read and approved the final manuscript.

## Pre-publication history

The pre-publication history for this paper can be accessed here:

http://www.biomedcentral.com/1471-2377/10/115/prepub
